# ADMIRE: analysis and visualization of differential methylation in genomic regions using the Infinium HumanMethylation450 Assay

**DOI:** 10.1186/s13072-015-0045-1

**Published:** 2015-12-01

**Authors:** Jens Preussner, Julia Bayer, Carsten Kuenne, Mario Looso

**Affiliations:** Bioinformatics Group, Max Planck Institute for Heart and Lung Research, Ludwigstrasse 43, 61231 Bad Nauheim, Germany

## Abstract

**Background:**

DNA methylation at cytosine nucleotides constitutes epigenetic gene regulation impacting cellular development and a wide range of diseases. Cytosine bases of the DNA are converted to 5-methylcytosine by the methyltransferase enzyme, acting as a reversible regulator of gene expression. Due to its outstanding importance in the epigenetic field, a number of lab techniques were developed to interrogate DNA methylation on a global range. Besides whole-genome bisulfite sequencing, the Infinium HumanMethylation450 Assay represents a versatile and cost-effective tool to investigate genome-wide changes of methylation patterns.

**Results:**

Analysis of DNA Methylation In genomic REgions (ADMIRE) is an open source, semi-automatic analysis pipeline and visualization tool for Infinium HumanMethylation450 Assays with a special focus on ease of use. It features flexible experimental settings, quality control, automatic filtering, normalization, multiple testing, and differential analyses on arbitrary genomic regions. Publication-ready graphics, genome browser tracks, and table outputs include summary data and statistics, permitting instant comparison of methylation profiles between sample groups and the exploration of methylation patterns along the whole genome. ADMIREs statistical approach permits simultaneous large-scale analyses of hundreds of assays with little impact on algorithm runtimes.

**Conclusions:**

The web-based version of ADMIRE provides a simple interface to researchers with limited programming skills, whereas the offline version is suitable for integration into custom pipelines. ADMIRE may be used via our freely available web service at https://bioinformatics.mpi-bn.mpg.de without any limitations concerning the size of a project. An offline version for local execution is available from our website or GitHub (https://github.molgen.mpg.de/loosolab/admire).

**Electronic supplementary material:**

The online version of this article (doi:10.1186/s13072-015-0045-1) contains supplementary material, which is available to authorized users.

## Background

Several epigenetic mechanisms control gene expression in cells [1]. One of these conserved mechanisms is DNA methylation, a process where cytosine bases of DNA are converted to 5-methylcytosine by the DNA methyltransferase (DNMT) enzymes. DNA methylation by these enzymes is a reversible regulator of gene expression. Methylated cytosine recruits proteins which are involved in gene repression and inhibit the binding of transcription factors. The pattern of DNA methylation in the genome undergoes changes during development and plays a role in a range of diseases, utilizing processes of de novo methylation and demethylation. In case of development and differentiation, differentiated cells display a stable, cell-type-specific methylation pattern, permanently switching off the expression of genes that are not essential for the respective cell type.

A number of lab techniques were developed to interrogate DNA methylation including whole-genome bisulfite sequencing (WGBS) and Infinium HumanMethylation450 Assays [2]. Although WGBS provides a comprehensive genome-wide coverage (around 28 million CpGs in humans), it is associated with relatively high costs for re-sequencing the whole genome. A similar method known as reduced representation bisulfite sequencing (RRBS) is intended to overcome this problem by sequencing just DNA fragments enclosing at least one CpG site. While Infinium HumanMethylation450 Assays reveal a less comprehensive picture compared to sequencing-based methods (approximately 0.5 million CpGs are addressed), economical factors render them highly attractive for epigenome-wide association studies (EWAS) involving up to thousands of individual samples [3] and represent an effective tool to identify biomarkers of disease states and progression [4].

Although Infinium HumanMethylation450 Assays are widely used, just very recently a cohort of noncommercial analysis pipelines was introduced. However, most of these tools are designed as command line tools. This is frequently accompanied with complex usage requirements which pose a significant challenge to researchers with limited programming skills. Furthermore, the genome-wide visualization of methylation sites, the visualization of significantly differentially methylated sites and downstream analyses have not been addressed optimally, yet. Here we introduce ADMIRE, an easy to use web-based tool intended to simplify usage inside a comprehensive application accessible by web interface as well as programmatically. ADMIRE generates publication-ready graphical overviews of differentially methylated loci and genome-wide overview tracks (Additional file 1) including advanced statistical methods to increase sensitivity. An included gene set enrichment analysis provides an overview on the entities that might link the significant sites.

## Results

### Comparison to existing software

Very recently, a cohort of noncommercial analysis pipelines was introduced and a current selection of widely used packages is reviewed in [5]. While the total number of tools intended to perform at least individual steps of HumanMethylation450 assay analysis is estimated to be around 20, only a minority is accessible via a graphical user interface and often limited to specific operating systems. A detailed comparison of tool features is listed in Additional file 2. An easy to use web-based application is only provided by RnBeads [6], although this might be the best way for biologists with limited programming skills to access an analysis pipeline. In contrast to RnBeads (restricted to 24 arrays), the web-based version of ADMIRE does not restrict the number of input arrays and was tested with a sample set of 689 arrays from a GEO dataset described below. Additionally, since calculation of per-probe test statistics is the main computational task (see algorithm description below), the runtime of ADMIRE is virtually independent of the number of input arrays. While most of the available tools provide functions for probe filtering and normalization, only a small number include functionality to create scalable visualizations or to detect differentially methylated positions and regions simultaneously. Furthermore, regions of interest are often pre-calculated and only a small number of tools allow statistics on individual regions of interest that can be provided by the user. Finally, none of the available tools provides a downstream analysis that is able to discover the linkage of differentially methylated genes. In order to generate a tool that combines all these critical features, we developed ADMIRE, a web-based tool for users without any computational background.

### ADMIREs calculation of test statistics

ADMIRE features five different normalization methods (see [7]) but can also work on raw methylation values. The pipeline performs two one-sided two-sample rank tests (Mann–Whitney *U* tests) based on the sample_group information provided. In contrast to the *t* test, the Mann–Whitney *U* test does not require normally distributed data. The one-sided two-sample tests are performed per Illumina probe on the array and between pairs of sample groups. Intentionally, two *p* values are obtained for each probe, indicating a higher probe methylation in a distinct group and allowing the subsequent combination of multiple single *p* values from within a genomic region of interest (tiles, promotors and the like) as suggested in [8]. The spatially correlated *p* values are combined with genomic regions by mapping probe specific *p* values onto pre-calculated or user-defined genomic regions, indicating no change or a higher methylation in either sample group. To create a *p* value for an entire region, the Stouffer–Liptak correction implemented in [9] is used. A 1-step Sidak correction for multiple testing is applied to obtain q-values (see [9]). In order to filter significantly differentially methylated regions, a user-defined q-value threshold is used.

### The web-based analysis platform

**Fig. 1:**
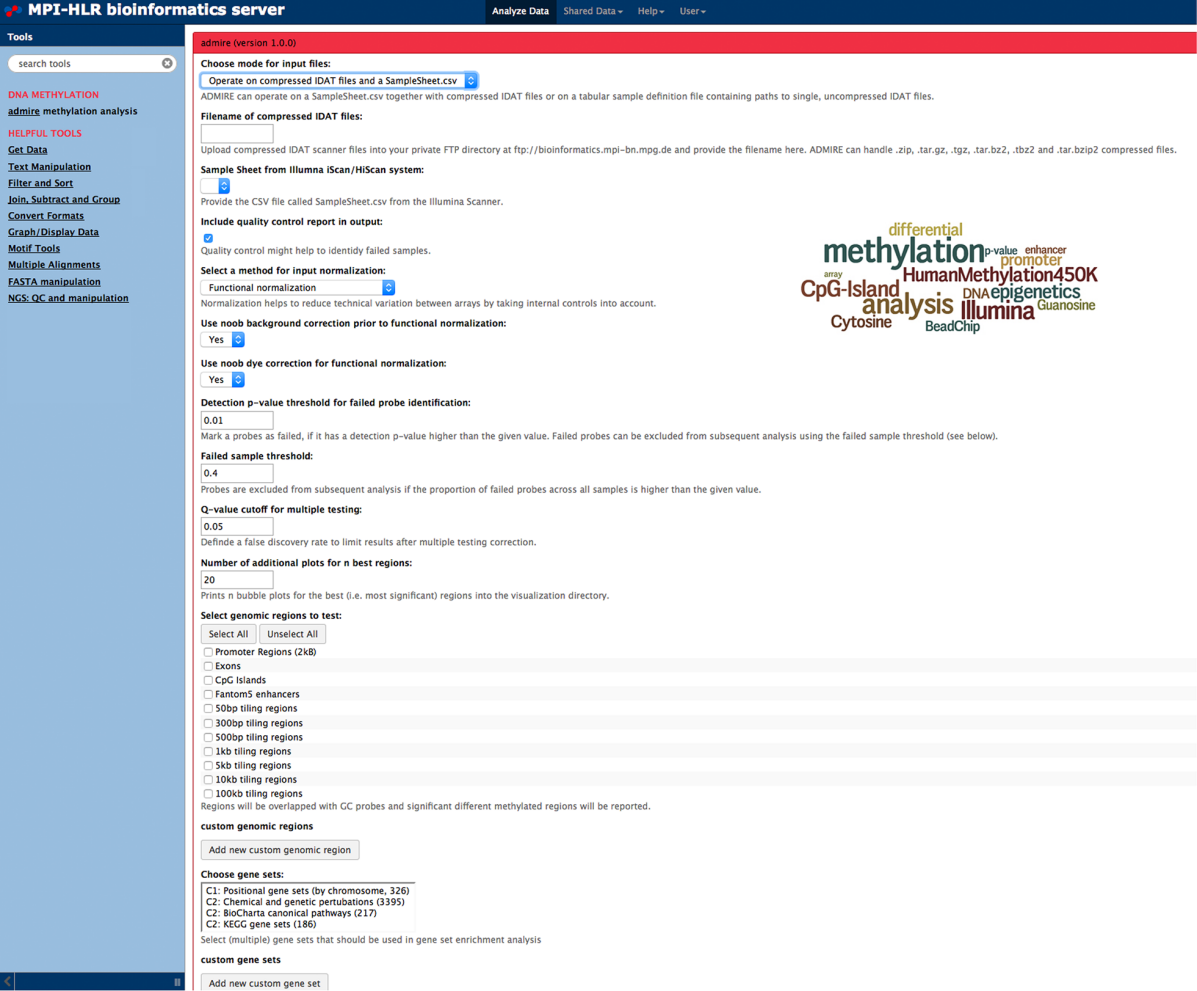
Graphical user interface for the ADMIRE pipeline. On the *left side* a set of helpful tools for file preparation and upload is listed. The *center pane* shows the ADMIRE parameters. Most parameters can be selected from drop down menus. Input and output files are listed in the *right pane*

The ADMIRE analysis platform is implemented as a web-based application (Fig. 1) and enables users with limited bioinformatics background to apply sophisticated methylation analysis. The web-based platform allows user accounts with the possibility to keep raw files and analyzed data in a workspace of unlimited size. The default output of a scanner system compatible to Illumina HumanMethylation450 Assay consists of a SampleSheet.csv file and a file directory named after the assays Sentrix-ID containing two compressed *.idat-files per sample. These raw files are supported by ADMIRE. Besides the original SampleSheet.csv, ADMIRE is also able to process a tab-separated sample definition file (see user manual, Additional file 3).

The settings file defines the groups that should be used for statistical testing. An all-vs-all comparison is performed with no limitation on the number of sample groups. Next, a wide range of analysis parameters can be adjusted, such as normalization method (SWAN, Functional, Quantile, Noob or Illumina), quality control filtering based on detection *p* values, failed sample threshold, Q-value cutoff for multiple testing as well as genomic regions for testing. A set of pre-calculated genomic regions are provided such as genome-wide tilings, annotations based on Gencode [10], as well as CpG islands and Fantom5 enhancers [11]. Furthermore, custom regions of interest can be uploaded to combine probes. To generate high-resolution graphics of differentially methylated regions, a numeric parameter is available to choose the number of graphics that will be generated from the most significantly altered regions. If the user is interested in a downstream analysis of differentially regulated regions, a gene set enrichment analysis can be performed on a selection of pre-defined gene sets [12] including chromosomal locations, pathways, diseases, and GO-terms. In addition to pre-defined sets, custom gene sets can be provided.

### Workflow

**Fig. 2:**
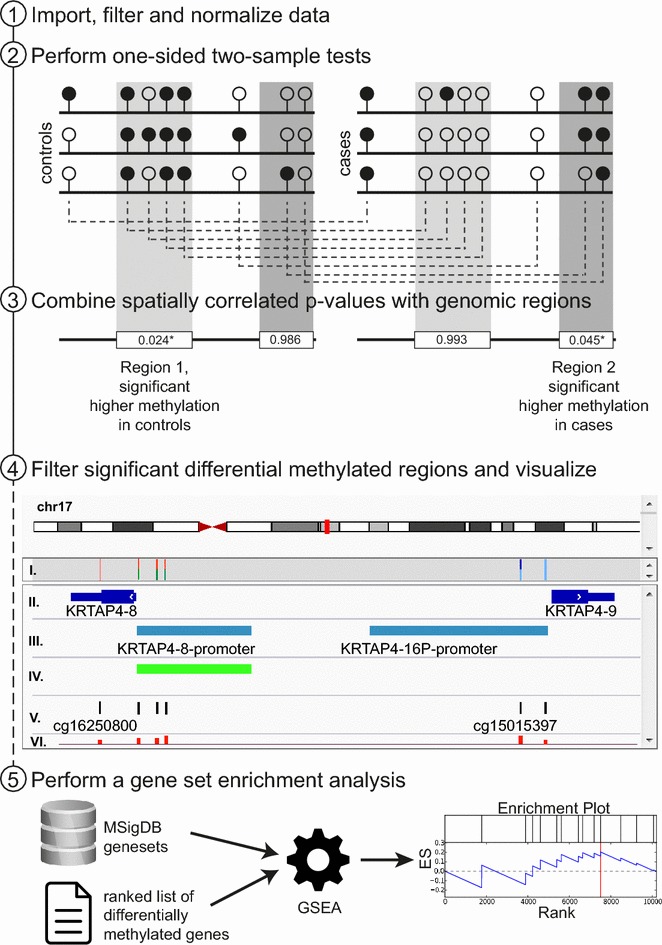
Workflow is illustrated on the *left side* as five steps. *Step 2* Controls and cases are illustrated as replicates with methylated (*black*) and unmethylated (*white*) CpG sites. Single sites are compared between controls and cases (*dashed lines*). *Step 3* Site-specific *p* values are combined into genomic regions and a representative *q* value is calculated for each region (*light gray*: higher methylation in control; *dark gray*: higher methylation in cases). *Step 4* IGV screenshot of array visualization; tracks represent: (*I.*) single CpG site *q* values for two conditions with a *color code*, (*II.*) positions of known genes, (*III.*), selected regions of interest, (*IV.*) significant regions found by the pipeline, (*V.*) all probes represented on the array, and (*VI.*) *bar plot* track denoting absolute methylation change (up/down). *Step 5* An optional gene set enrichment analysis (GSEA) can be performed using pre-defined or custom gene sets and ranked lists of differentially methylated genes

Once the analysis is started, ADMIRE evaluates the sample definition file and prints out an error message in case files are missing or cannot be read. The raw files are preprocessed and filtered by the functions from the R package *minfi* [7], according to the parameters set. Aggregated data is used to generate a quality control report in PDF format and normalized beta and m values are provided as tabular data (Fig. 2, step 1). In accordance to the groups defined earlier, all-vs-all pairwise comparisons of per-probe methylation are performed automatically. To call the significant differences in terms of methylation, ADMIRE performs statistical tests as described in the section above (Fig. 2, step 2).

Next, spatially correlated *p* values are combined with respect to the genomic regions defined by the user [9]. The generated result list includes all genomic regions, sorted by significance of methylation changes between the groups specified and the min/max/median change of methylation rate is calculated for further filtering (Fig. 2 step 3). For the most significant differentially methylated regions, a high-resolution image is generated (see Additional file 1). Finally, all results are transformed into BED format data tracks to allow visualization of differentially methylated regions in commonly used genome viewers such as IGV [13] or UCSC [14] (Fig. 2, step 4). Additionally, the output includes comma-separated tables that can be used to filter for specific genes, genomic locations, coverage, min/max/median change, *p* values, and/or *q* values. Details on the output files can be found in the methods section and in Additional file 3. Given that regions with a direct link to genes (indicated by a *gene_name* property) were chosen as regions of interest, a gene set enrichment analysis can be performed [12]. The enrichment analysis calculates an enrichment score (ES) for each gene set, depending on the ranks and differences in methylation of genes that are members of the gene set. In combination with graphs for enrichment score calculations, it can be inferred whether higher methylation in controls or cases contributed most to the enrichment of the gene set. Additionally, a heat map graphically represents a leading edge analysis that allows the detection of gene sets with a high overlap of core genes that mainly affect the ES (Fig. 2, step 5). All results listed above are generated in the workspace and can be downloaded as individual files or as a compressed archive from the web-based platform.

### Performance evaluation and comparison to the existing gold standard

**Fig. 3:**
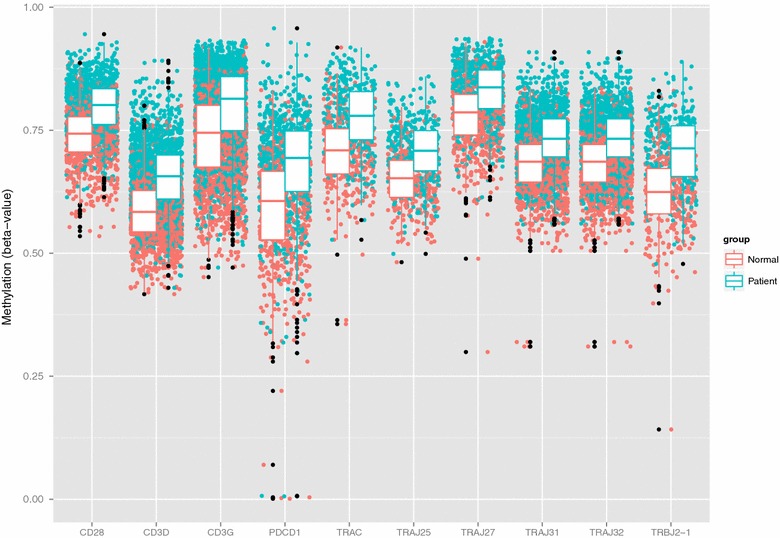
Methylation of members of the T-cell receptor signaling pathway in rheumatic arthritis (*Patients*) vs. healthy individuals (*Normal*). Shown are the beta values from all methylation sites of 2-kb upstream regulatory regions across all 689 replicates. *Black dots* represent *boxplot outliers*

To demonstrate the ease of use, the robustness and applicability of ADMIRE, we downloaded 689 HumanMethylation450 Assay samples from a study analyzing DNA methylation as an intermediary of genetic risk in rheumatoid arthritis (GEO GSE42861) [15]. ADMIRE was invoked from the web interface using a custom sample-definition file (see “Methods”) with default parameters. We selected all 2-kB promoter regions and chose positional gene sets as input for the enrichment analysis. Since the runtime of ADMIRE is virtually independent of input size, the results were obtained after 24 h with a maximum memory usage of 65 GB RAM. As the analysis in [15] was performed on single methylation sites and we did not intent to replicate the analysis, validation was done via an unbiased gene set enrichment analysis using positional gene sets as input. We identified the constant (*TRAC*) and variable (*TRAV*/*TRAJ*) segments of the T-cell receptor alpha chain on chr14q11 locus as higher methylated in arthritis patients. Additionally, four known members of the T-cell receptor signaling pathway, *CD28*, *CD3G*, *CD3D* as well as *PDCD1*, were found to be higher methylated in patients (Fig. 3).

**Fig. 4:**
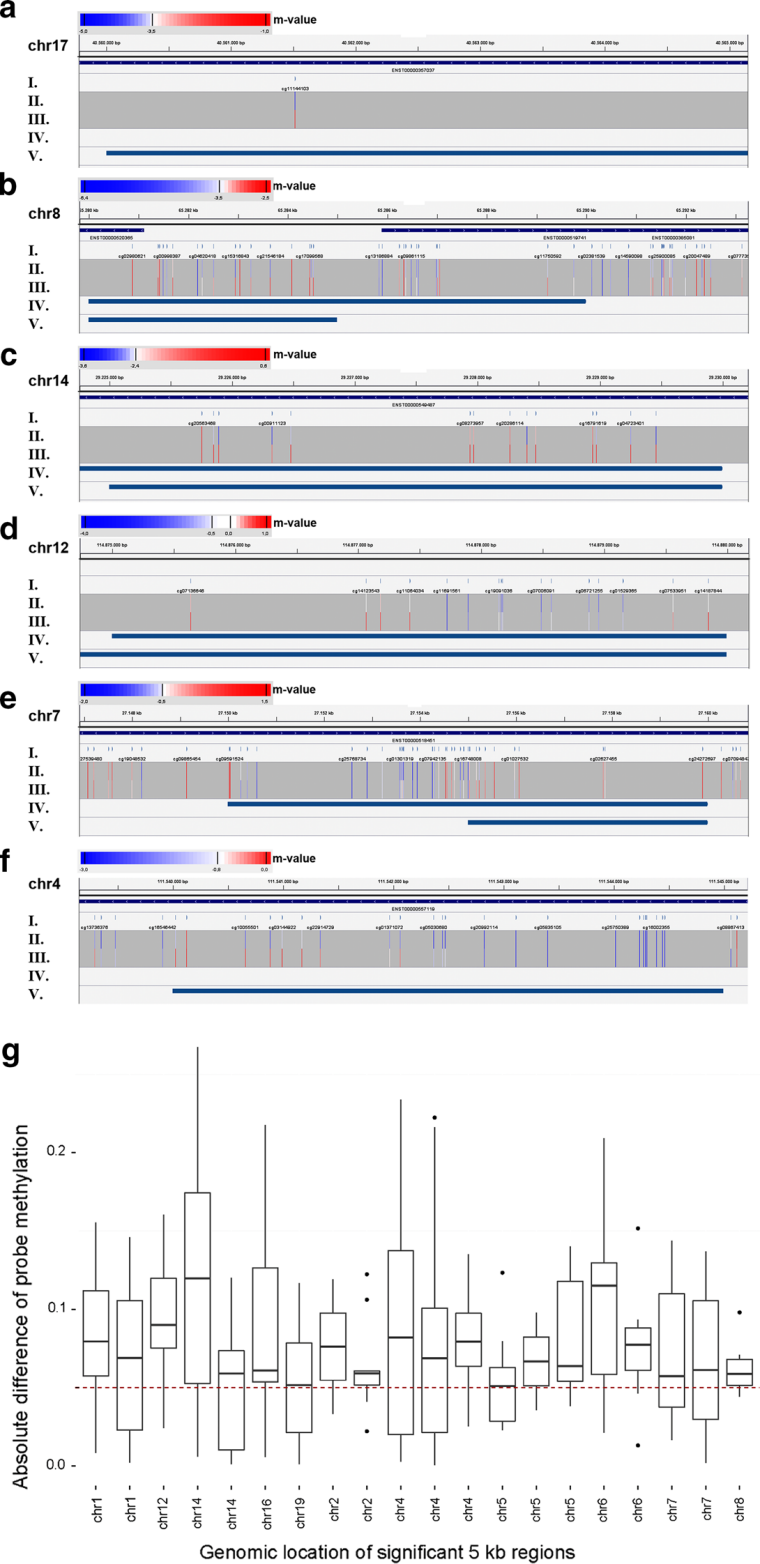
IGV screenshots showing methylation across several genomic locations and *boxplots* for all significant sites. **a**–**f** Tracks shown are as follows: *I.* Methylation sites present on the HumanMethylation450 K Chip, *II.* *Color-coded* methylation values from control samples, *III.* Color-coded methylation values from AF samples, *IV.* Differentially methylated 10-kbp tiling regions called by ADMIRE, *V.* Differentially methylated 5-kbp tiling regions called by ADMIRE. The *color bar* encodes the *m* value, with blue indicating low methylation values and *red* indicating high methylation values. The absolute scale is created indvidually for each bar. Track *IV* and *V* are only used if the search with the corresponding input (5- or 10-kB tiling size) resulted in a significant region. **a** A 5-kbp region from chr17 called to be differentially methylated by RnBeads with an adjusted *p* value of 0.00008. **b**–**f** Top 5 differentially methylated regions from Admire with *q* values between 0.0004 and 0.003. **g** *Boxplots* for 20 significantly changed protein coding genes (higher in AF sample) identified by ADMIRE. *Each box* illustrates the distribution of absolute differences of the methylation values in the respective significantly changed region (see also Additional file 4). The cutoff at median methylation value of 5 % is shown as *red dashed* *line*

In order to compare ADMIRE to RnBeads, the current gold standard for HumanMethylation450 Assay analysis, we used an additional dataset of smaller size since the RnBeads [16] web interface is restricted to 24 samples. Our test dataset contains 11 samples from a study analyzing permanent atrial fibrillation (GEO GSE62727). This dataset was analyzed by RnBeads using default parameters (5-kB pre-calculated tiling regions) as well as the ADMIRE pipeline. To match the output from RnBeads and enable a direct comparison, we selected all 5-kB tiling regions as input for ADMIRE (see “Methods”). Our tool found twenty 5-kB regions corresponding to protein coding genes to be higher methylated in fibrillating atria (see Additional file 4) with a median methylation change of up to 12 %. Next, we carried out a second run with ADMIRE using 10-kB tiling regions as input to test for reproducibility of statistically significantly changed regions. Besides nine genes present in both result files, another 14 genes were identified from 10-kB regions only, with a median methylation change up to 45 % (see Additional file 5). RnBeads identified only one region to be higher methylated in fibrillating atria. This genomic location was not reported by ADMIRE. Some representative significant regions found by ADMIRE and the single region found by RnBeads are shown in Fig. 4a–f. We chose an indirect way to evaluate specificity and significance of regions reported by ADMIRE but not by RnBeads. To evaluate the latter, we visualized the homogeneity of the methylation change over all 5-kB tiling regions detected by ADMIRE in Fig. 4g. The boxplots represent all single methylation sites, combined in accordance to the tiling region. Their level and spread present a global overview in order to investigate the magnitude of the methylation changes. The user can interpret this information to select an appropriate threshold. To evaluate the specificity of our findings, we performed a functional analysis. This showed an enrichment of transcriptional regulation, driven by transcription factors such as HOX A, TBX5, and PITX2 (Additional file 6). This is remarkable, as initial GWAS studies identified a major risk region where the presence of a variant increased the risk of AF up to 65 %. Located proximally to the variant, PITX2 is a transcription factor import for cardiogenesis, especially for left–right signaling and L/R atrial identity. Knockout of PITX2 lead to a shortened atrial action potential in haploinsufficient mice and increased the susceptibility to AF [17]. Expression analysis identified the Sinoatrial node (SAN) specific genes Shox2, Tbx3, and Hcn4 as upregulated in PITX2 null-mutant embryos [18]. A recent study additionally identified two microRNAs miR-17-92 and miR-106b-25 as direct targets of PITX2 that can repress Shox2 and Tbx3 upon transcription [19] and promote the expression of Cx43, a connexin protein forming gap junctions that allow the interchange of charged ions between adjacent cells [20]. Another GWAS study linked TBX5 to AF [21]. The homeobox transcription factor may play a role in heart development and specification of limb identity [22]. Interestingly, TBX5 was identified as interactor of Tbx3, a regulator of the SAN gene program [23]. Hoxa3 is another important gene in heart chamber morphogenesis, since Hoxa3-expressing progenitor cells in the second heart field give rise to the atria and parts of the outflow tract [24].

Summarizing these findings, we conclude that using genome-wide tiling regions as well as the positional gene sets in the implemented gene set enrichment provide a powerful and yet unbiased downstream analysis option to the user. As shown by the comparison to RnBeads, we assume ADMIRE to have a higher sensitivity to detect small changes in methylation rate, as the user can decide upon appropriate thresholds for absolute difference in methylation. Both datasets used for performance evaluation are available as shared data libraries on the ADMIRE web server (see Additional file 3 for loading shared data libraries).

## Discussion

Integration and differential analysis of DNA methylation represents a major topic in clinical bioinformatics, most often addressed by whole-genome bisulfite sequencing or Infinium HumanMethylation450 Assays. Given the nature of methylation assay data, most of the analysis tools developed in the past are primarily focused on command line-based programming libraries, such as the R-based ChAMP [25] or minfi [7] packages, limiting the use of these tools to users with at least some programming skills. A second group of tools are intended to provide a comprehensive graphical interface to the user, including MethLAB [26], COHCAP [27], EpiDiff [28], and the Genome Studio (Illumina, proprietary license). Within this group, only two tools are available (RnBeads and ADMIRE) that are capable to provide their service not only on the command line but also as a web-based graphical user interface. While all of these programs are arguably valuable contributions to facilitate the analysis of Illumina HumanMethylation450 Assays, many may be too demanding to wet lab researchers and clinicians with limited computational skills. To face these needs, a web frontend might impose the least number of restrictions to the user. The intuitive, interactive, and relatively simple interface of ADMIRE facilitates the upload, analysis, and visualization of a complex technology. The input is limited to the raw files, a sample sheet describing the groups of interest and the selection of a few parameters. Common experimental setups in molecular studies that define more than two groups are addressed by automated all-vs-all comparisons. Genomic regions and gene sets are available as precomputed files, but the possibility to upload custom files offers a variety of downstream analysis options. Unfortunately, public web services frequently perform very limited in terms of throughput, since the workload has to be managed by the website provider. In case of HumanMethylation450Assays, the web-based analysis from RnBeads is limited to 24 arrays. In contrast, the algorithm of ADMIRE is designed to transfer the computational effort to the number of probes that are tested and is influenced only in a minor grade by the number of arrays under investigation. This focus permits the provision of the web service not only for small projects with a limited number of arrays, but also for large projects encompassing hundreds of input samples (performance evaluation with 689 input samples). Results from the original publication [15] handling these arrays, identify the MHC region as a major genetic risk loci in rheumatic arthritis. MHC peptides are bound by T-cell receptors together with their co-receptors *CD28* and *CD3*. ADMIRE highly supports this result, by linking differential methylation in the T-cell receptor signaling pathway as an alternative mechanism to rheumatic arthritis. Furthermore, the differential methylation of *PDCD1* (*PD*-*1*), a co-inhibitor of the T-cell receptor signaling pathway involved in T-cell activation [29] could represent another mechanism by disturbing the control of autoimmunity.

## Conclusion

ADMIRE offers an intuitive interface to analyze DNA methylation patterns based on Infinium HumanMethylation450 Assays. Whereas most existing analysis tools are designed to be used on the command line, ADMIRE provides an easy to use web-based service as well as a version for local execution. A wide range of experimental and statistical settings can be adjusted, including normalization methods and detection of differentially methylated positions and regions. Whereas these regions are often pre-calculated in other tools, ADMIRE can calculate statistics on individual regions of interest provided by the user. As an optional step towards downstream analysis, ADMIRE additionally implements a gene set enrichment procedure. ADMIRE is freely accessible without a limit on experimental size at https://bioinformatics.mpi-bn.mpg.de.

## Methods

### Implementation

ADMIRE was implemented in Bash, R, and Python while making use of the open-source Bioconductor package minfi [7] and the comb-p [9] tool for data processing. Additionally, a variant of GSEA [12] is fully implemented in ADMIRE for gene set enrichment analysis. The pipeline was integrated into a Galaxy-based [30] platform similar to MIRPIPE [31] to provide online access but is also available for download and local execution. Input data can either be used immediately from Infinium HumanMethylation450 Assay compatible scanner systems (*SampleSheet.csv* and *.idat-files) or the sample file can be prepared as a tab-separated text file. A detailed explanation of all input and output files is available in Additional file 3.

### Generation of genetic regions and gene sets

Gene information from the GENCODE V19 [10] annotation was used to extract genomic regions for all exons (GTF feature type *exon*) and all 2-kB promoter regions downstream of the TSS. CpG islands were extracted from the Bioconductor annotation package *IlluminaHumanMethylation450kanno.ilmn12.hg19*. Enhancer information was downloaded from the Fantom5 project web site [11]. Bedtools *makewindows* function was used to generate genome-wide tiling regions of different sizes ranging from 50 bp up to 100 kB. All genomic regions were saved as bed files, keeping the *gene_name* property, if applicable. Gene sets for gene set enrichment analysis were downloaded from MSigDB [12] and are contained in the distribution of ADMIRE.

### Benchmark and analysis of publicly available datasets

All raw *.idat-files were downloaded from the respective GEO project site (GSE42861 and GSE62727). Tabular sample definition files were generated (see user manual). Admire was invoked using default parameters and the following genomic regions and gene sets: 2-kB promoter regions and positional gene sets for the rheumatic arthritis (RA) data and 5- and 10-kB genomic tiling regions for the atrial fibrillation (AF) data. Results from the RA data were limited to contain only protein coding genes and TR_C/TR_J genes with a Q-value below 0.01 and an absolute median difference in methylation between normal and patient samples of 5 % (Additional file 7). Remaining genes with higher methylation in patients were subjected to a GO analysis with two unranked lists of genes using GORILLA [32] (Additional file 8) and methylation values for significantly altered genes that map to the T-cell receptor signaling pathway were plotted in Fig. 3. Results from the AF data (Additional file 4) were annotated with their nearest gene using bedtools closest function and were limited to contain only protein coding genes with a median absolute difference of 5 %. Gene names were subjected to a GO analysis as described above. To analyze the sensitivity of ADMIRE, per-probe absolute differences were extracted using bedtools map function and plotted per chromosomal location in Fig. 4g.

## Additional files

10.1186/s13072-015-0045-1 Examples of publication ready graphical overviews.
10.1186/s13072-015-0045-1 Comparison of available tools and packages for analysis of Illumina HumanMethylation450 Assays.
10.1186/s13072-015-0045-1 ADMIRE documentation. The documentation provides description of all available parameters, input and output files as well as an example analysis of the atrial fibrillation data used in this publication.
10.1186/s13072-015-0045-1 Significantly differentially methylated tiling regions in AF. Tiling regions were annotated with their nearest gene (see Methods).
10.1186/s13072-015-0045-1 Absolute difference of methylation in 5 and 10 kB tiling regions in atrial fibrillation reported by ADMIRE. Boxplots give information about the magnitude of methylation change.
10.1186/s13072-015-0045-1 Beta values of protein coding genes with significantly differential methylation between patients with atrial fibrillation and healthy individuals.
10.1186/s13072-015-0045-1 Significantly differentially methylated promoter regions in RA.
10.1186/s13072-015-0045-1 Enriched functional GO-Terms of genes with higher methylation values in RA. Background color codes for *p* values in the following way: >10–3 (white), 10–3 to 10–5 (light yellow) and 10–7 to 10–9 (orange).

## Abbreviations

CpG: cytosine-phosphate-Guanine
DNA: deoxyribonucleic acid
GEO: gene expression omnibus database
GO: gene ontology
GTF: general transfer format
GWAS: genome-wide association study
IGV: integrative genomics viewer
kB: kilo basepairs
MHC: major histocompatibility complex
RAM: random access memory
SWAN: subset-quantile within array normalization
UCSC: University of California, Santa Cruz
