# Supplementary material for: ADMIRE: analysis and visualization of differential methylation in genomic regions using the Infinium HumanMethylation450 Assay
Source: Epigenetics Chromatin. 2015 Dec 1;8:51. doi: 10.1186/s13072-015-0045-1 (PMC4666223; doi:10.1186/s13072-015-0045-1)
Supplement: Supplementary file 1 — 10.1186/s13072-015-0045-1 Examples of publication ready graphical overviews. [file 13072_2015_45_MOESM1_ESM.pdf]

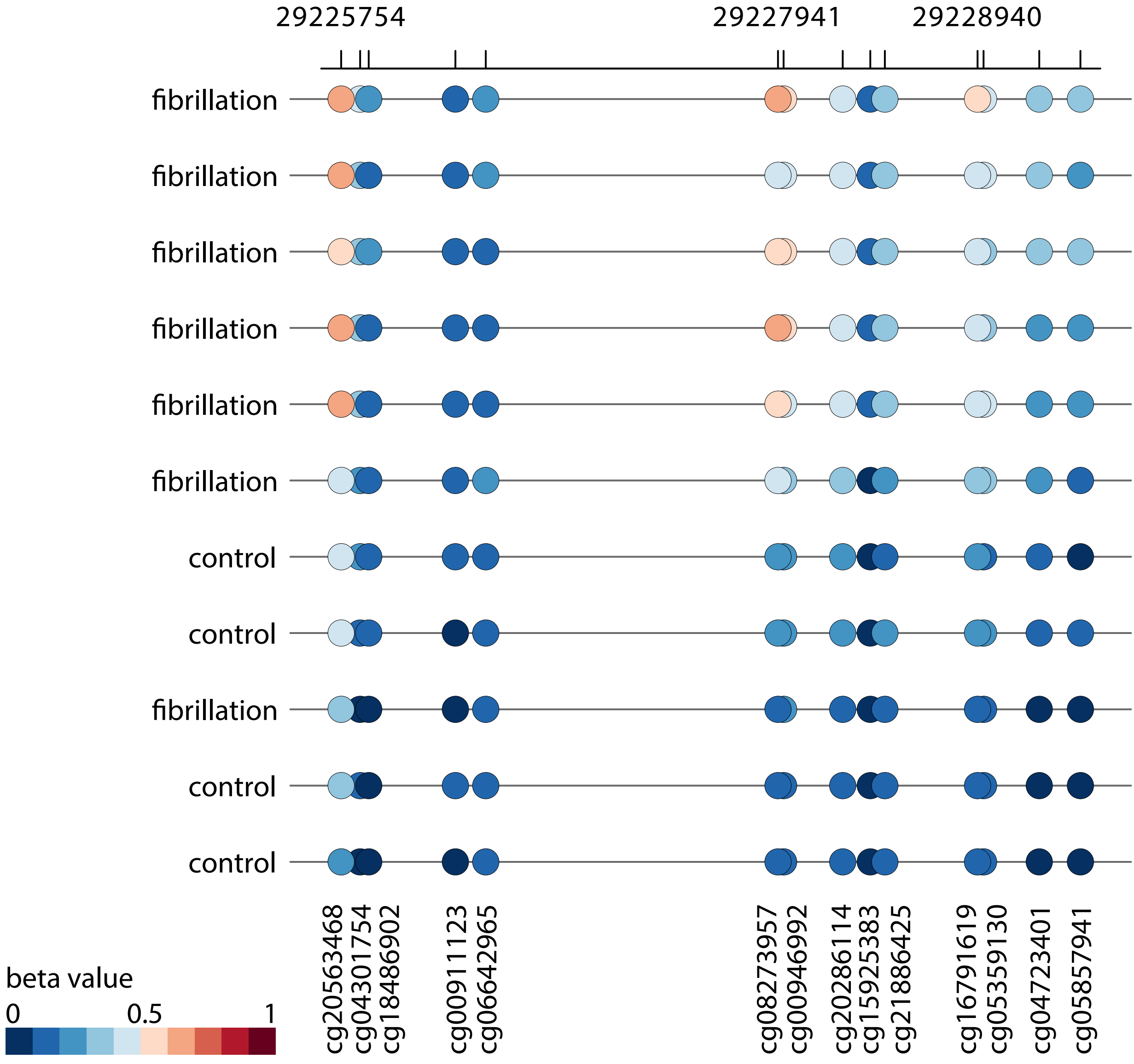
29225754
29227941
29228940
fibrillation
fibrillation
fibrillation
fibrillation
fibrillation
fibrillation
control
control
fibrillation
control
control
cg20563468
cg04301754
cg18486902
cg00911123
cg06642965
cg08273957
cg00946992
cg20286114
cg15925383
cg21886425
cg16791619
cg05359130
cg04723401
cg05857941
beta value
0
0.5
1

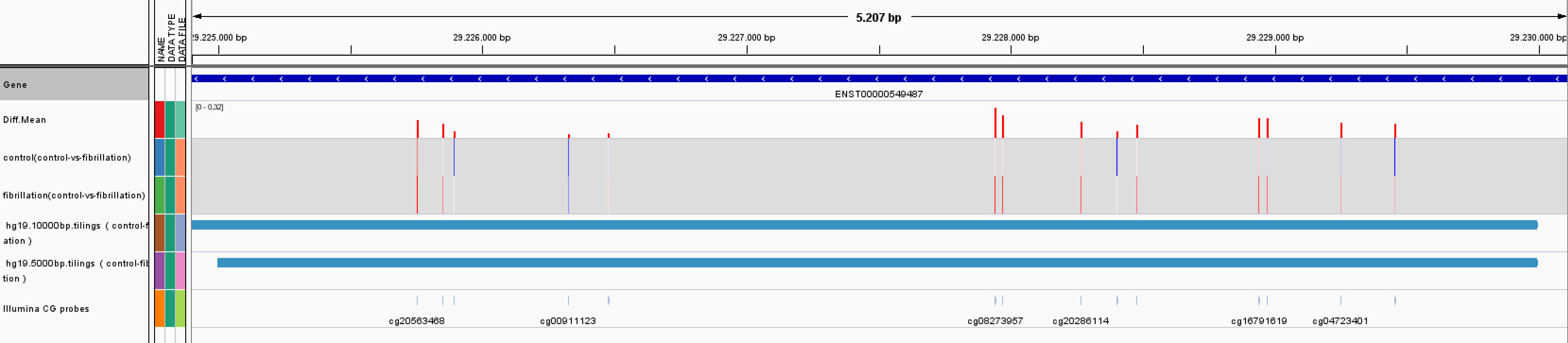

5.207 bp
29.225.000 bp
29.226.000 bp
29.227.000 bp
29.228.000 bp
29.229.000 bp
29.230.000 bp
NAME
DATA TYPE
DATA FILE
Gene
ENST00000549487
Diff.Mean
[0 - 0,32]
control(control-vs-fibrillation)
fibrillation(control-vs-fibrillation)
hg19.10000bp.tilings (control-f ation)
hg19.5000bp.tilings (control-fib tion)
Illumina CG probes
cg20563468
cg00911123
cg08273957
cg20286114
cg16791619
cg04723401

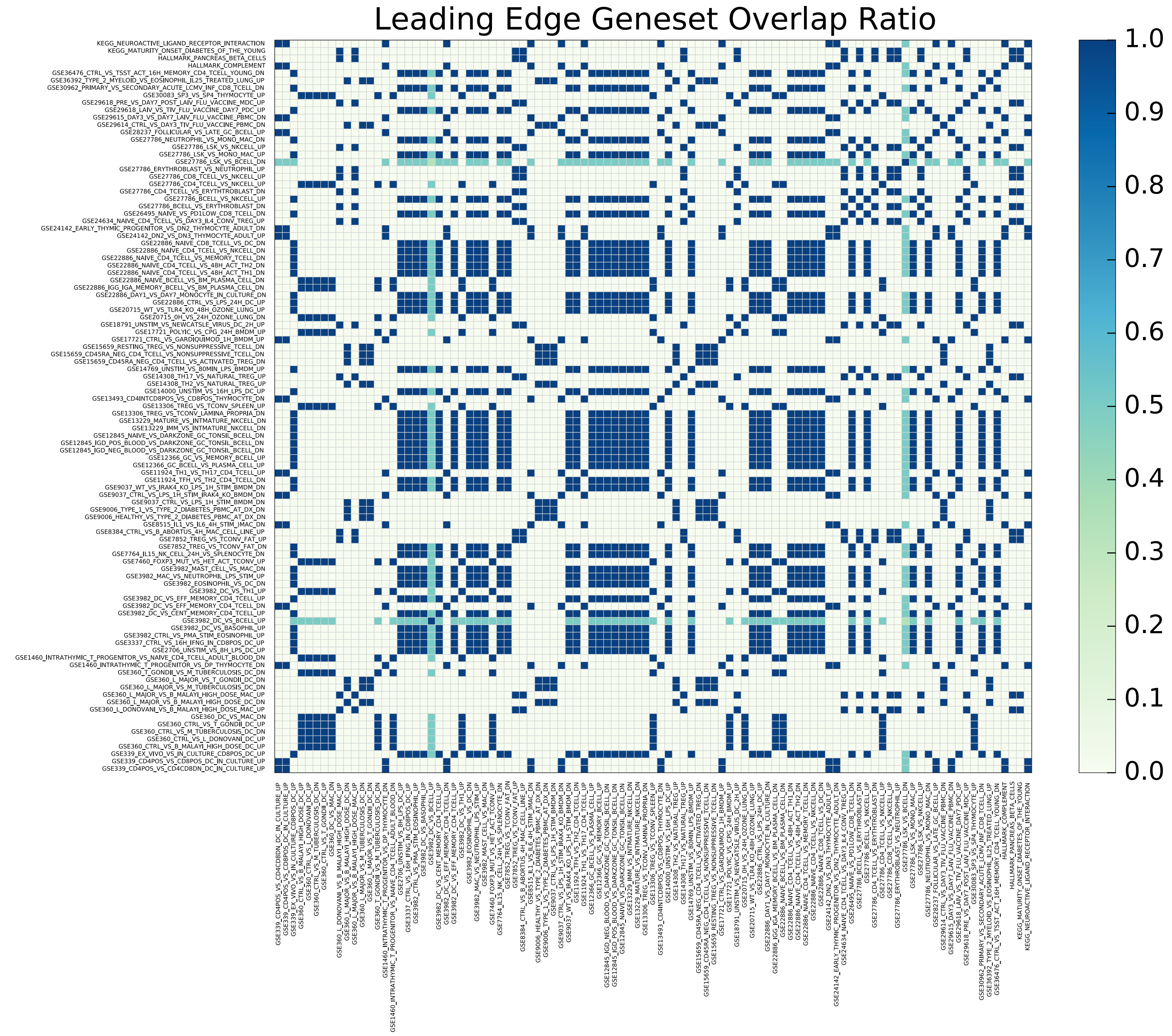
Leading Edge Geneset Overlap Ratio
KEGG_NEUROACTIVE_LIGAND_RECEPTOR_INTERACTION
KEGG_MATURITY_ONSET_DIABETES_OF_THE_YOUNG
HALLMARK_PANCREAS_BETA_CELLS
HALLMARK_COMPLEMENT
GSE36476_CTRL_VS_TSST_ACT_16H_MEMORY_CD4_TCELL_YOUNG_DN
GSE36392_TYPE_2_MYELOID_VS_EOSINOPHIL_IL25_TREATED_LUNG_UP
GSE30962_PRIMARY_VS_SECONDARY_ACUTE_LCMV_INF_CD8_TCELL_DN
GSE30083_SP3_VS_SP4_THYMOCYTE_UP
GSE29618_PRE_VS_DAY7_POST_LAIV_FLU_VACCINE_MDC_UP
GSE29618_LAIV_VS_TIV_FLU_VACCINE_DAY7_PDC_UP
GSE29615_DAY3_VS_DAY7_LAIV_FLU_VACCINE_PBMC_DN
GSE29614_CTRL_VS_DAY3_TIV_FLU_VACCINE_PBMC_DN
GSE28237_FOLLICULAR_VS_LATE_GC_BCELL_UP
GSE27786_NEUTROPHIL_VS_MONO_MAC_DN
GSE27786_LSK_VS_NKCELL_UP
GSE27786_LSK_VS_MONO_MAC_UP
GSE27786_LSK_VS_BCELL_DN
GSE27786_ERYTHROBLAST_VS_NEUTROPHIL_UP
GSE27786_CD8_TCELL_VS_NKCELL_UP
GSE27786_CD4_TCELL_VS_NKCELL_UP
GSE27786_CD4_TCELL_VS_ERYTHROBLAST_DN
GSE27786_BCELL_VS_NKCELL_UP
GSE27786_BCELL_VS_ERYTHROBLAST_DN
GSE26495_NAIVE_VS_PD1LOW_CD8_TCELL_DN
GSE24634_NAIVE_CD4_TCELL_VS_DAY3_IL4_CONV_TREG_UP
GSE24142_EARLY_THYMIC_PROGENITOR_VS_DN2_THYMOCYTE_ADULT_DN
GSE24142_DN2_VS_DN3_THYMOCYTE_ADULT_UP
GSE22886_NAIVE_CD8_TCELL_VS_DC_DN
GSE22886_NAIVE_CD4_TCELL_VS_NKCELL_DN
GSE22886_NAIVE_CD4_TCELL_VS_MEMORY_TCELL_DN
GSE22886_NAIVE_CD4_TCELL_VS_48H_ACT_TH2_DN
GSE22886_NAIVE_CD4_TCELL_VS_48H_ACT_TH1_DN
GSE22886_NAIVE_BCELL_VS_BM_PLASMA_CELL_DN
GSE22886_IGG_IGA_MEMORY_BCELL_VS_BM_PLASMA_CELL_DN
GSE22886_DAY1_VS_DAY7_MONOCYTE_IN_CULTURE_DN
GSE22886_CTRL_VS_LPS_24H_DC_UP
GSE20715_WT_VS_TLR4_KO_48H_OZONE_LUNG_UP
GSE20715_0H_VS_24H_OZONE_LUNG_DN
GSE18791_UNSTIM_VS_NEWCATSLE_VIRUS_DC_2H_UP
GSE17721_POLYIC_VS_CPG_24H_BMDM_UP
GSE17721_CTRL_VS_GARDIQUIMOD_1H_BMDM_UP
GSE15659_RESTING_TREG_VS_NONSUPPRESSIVE_TCELL_DN
GSE15659_CD45RA_NEG_CD4_TCELL_VS_NONSUPPRESSIVE_TCELL_DN
GSE15659_CD45RA_NEG_CD4_TCELL_VS_ACTIVATED_TREG_DN
GSE14769_UNSTIM_VS_80MIN_LPS_BMDM_UP
GSE14308_TH17_VS_NATURAL_TREG_UP
GSE14308_TH2_VS_NATURAL_TREG_UP
GSE14000_UNSTIM_VS_16H_LPS_DC_UP
GSE13493_CD4INTCD8POS_VS_CD8POS_THYMOCYTE_DN
GSE13306_TREG_VS_TCONV_SPLEEN_UP
GSE13306_TREG_VS_TCONV_LAMINA_PROPRIA_DN
GSE13229_MATURE_VS_INTMATURE_NKCELL_DN
GSE13229_IMM_VS_INTMATURE_NKCELL_DN
GSE12845_NAIVE_VS_DARKZONE_GC_TONSIL_BCELL_DN
GSE12845_IGD_POS_BLOOD_VS_DARKZONE_GC_TONSIL_BCELL_DN
GSE12845_IGD_NEG_BLOOD_VS_DARKZONE_GC_TONSIL_BCELL_DN
GSE12366_GC_VS_MEMORY_BCELL_UP
GSE12366_GC_BCELL_VS_PLASMA_CELL_UP
GSE11924_TH1_VS_TH17_CD4_TCELL_UP
GSE11924_TFH_VS_TH2_CD4_TCELL_DN
GSE9037_WT_VS_IRAK4_KO_LPS_1H_STIM_BMDM_DN
GSE9037_CTRL_VS_LPS_1H_STIM_IRAK4_KO_BMDM_DN
GSE9037_CTRL_VS_LPS_1H_STIM_BMDM_DN
GSE9006_TYPE_1_VS_TYPE_2_DIABETES_PBMC_AT_DX_DN
GSE9006_HEALTHY_VS_TYPE_2_DIABETES_PBMC_AT_DX_DN
GSE8515_IL1_VS_IL6_4H_STIM_)MAC_DN
GSE8384_CTRL_VS_B_ABORTUS_4H_MAC_CELL_LINE_UP
GSE7852_TREG_VS_TCONV_FAT_UP
GSE7852_TREG_VS_TCONV_FAT_DN
GSE7764_IL15_NK_CELL_24H_VS_SPLENOCYTE_DN
GSE7460_FOXP3_MUT_VS_HET_ACT_TCONV_UP
GSE3982_MAST_CELL_VS_MAC_DN
GSE3982_MAC_VS_NEUTROPHIL_LPS_STIM_UP
GSE3982_EOSINOPHIL_VS_DC_DN
GSE3982_DC_VS_TH1_UP
GSE3982_DC_VS_EFF_MEMORY_CD4_TCELL_UP
GSE3982_DC_VS_EFF_MEMORY_CD4_TCELL_DN
GSE3982_DC_VS_CENT_MEMORY_CD4_TCELL_UP
GSE3982_DC_VS_BCELL_UP
GSE3982_DC_VS_BASOPHIL_UP
GSE3982_CTRL_VS_PMA_STIM_EOSINOPHIL_UP
GSE3337_CTRL_VS_16H_IFNG_IN_CD8POS_DC_UP
GSE2706_UNSTIM_VS_8H_LPS_DC_UP
GSE1460_INTRATHYMIC_T_PROGENITOR_VS_NAIVE_CD4_TCELL_ADULT_BLOOD_DN
GSE1460_INTRATHYMIC_T_PROGENITOR_VS_DP_THYMOCYTE_DN
GSE360_T_GONDII_VS_M_TUBERCULOSIS_DC_DN
GSE360_L_MAJOR_VS_T_GONDII_DC_DN
GSE360_L_MAJOR_VS_M_TUBERCULOSIS_DC_DN
GSE360_L_MAJOR_VS_B_MALAYI_HIGH_DOSE_MAC_UP
GSE360_L_MAJOR_VS_B_MALAYI_HIGH_DOSE_DC_DN
GSE360_L_DONOVANI_VS_B_MALAYI_HIGH_DOSE_MAC_UP
GSE360_DC_VS_MAC_DN
GSE360_CTRL_VS_T_GONDII_DC_UP
GSE360_CTRL_VS_M_TUBERCULOSIS_DC_DN
GSE360_CTRL_VS_L_DONOVANI_DC_UP
GSE360_CTRL_VS_B_MALAYI_HIGH_DOSE_DC_UP
GSE339_EX_VIVO_VS_IN_CULTURE_CD8POS_DC_UP
GSE339_CD4POS_VS_CD8POS_DC_IN_CULTURE_UP
GSE339_CD4POS_VS_CD4CD8DN_DC_IN_CULTURE_UP
1.0
0.9
0.8
0.7
0.6
0.5
0.4
0.3
0.2
0.1
0.0

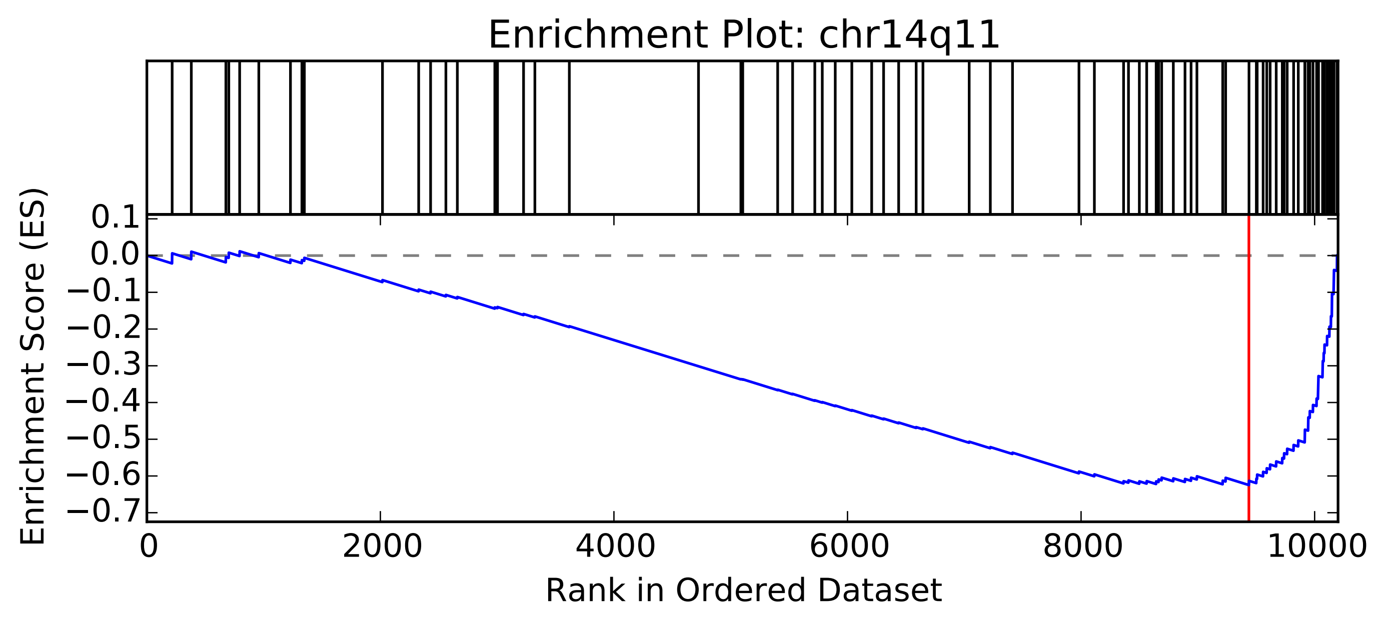

Enrichment Plot: chr14q11
Enrichment Score (ES)
0.1
0.0
−0.1
−0.2
−0.3
−0.4
−0.5
−0.6
−0.7
0
2000
4000
6000
8000
10000
Rank in Ordered Dataset
