# Supplementary material for: ADMIRE: analysis and visualization of differential methylation in genomic regions using the Infinium HumanMethylation450 Assay
Source: Epigenetics Chromatin. 2015 Dec 1;8:51. doi: 10.1186/s13072-015-0045-1 (PMC4666223; doi:10.1186/s13072-015-0045-1)
Supplement: Supplementary file 3 — 10.1186/s13072-015-0045-1 ADMIRE documentation. The documentation provides description of all available parameters, input and output files as well as an example analysis of the atrial fibrillation data used in this publication. [file 13072_2015_45_MOESM3_ESM.zip › custom/index.html]

  


Custom input - ADMIRE


ADMIRE

- - Home
  - - - Using the web service
      - Analysing example datasets
      - Analysing custom datasets
      - Available parameters- - - Command-line usage
          - Installation
          - HiScan/iScan scanner files
          - Custom input
          - Genomic regions
          - Gene sets
          - Available parameters- - - Output
              - - - MIT License

ADMIRE

- Docs »
- Command-line usage »
- Custom input
- Edit on GitHub

---

ADMIRE is also able to process a tab-separated sample definition file, with the following columns:

| column | explanation |
| --- | --- |
| sample\_id | an arbitrary sample identifier. Note that corresponding green and red channel files need the same identifier. |
| file | relative or absolute path of a red or green channel idat file. |
| channel | indicates whether the file is from the red or green channel (can be *Red* or *Grn*). |
| sample\_group | an arbitrary string identifying the sample group of the sample. |

For example, the sample definition file could look like:

```
sample_id   file    channel sample_group
1   8769527070/8769527070_R01C01_Grn.idat   Grn control
1   8769527070/8769527070_R01C01_Red.idat   Red control
2   8769527070/8769527070_R01C02_Grn.idat   Grn treatment
3   8769527070/8769527070_R02C01_Red.idat   Red treatment
```

ADMIRE can then be called with `admire -s sample_definition.txt` and will look for the \*.idat files specified in the sample definition file.

Next 
 Previous

---

Built with MkDocs using a theme provided by Read the Docs.

GitHub
« Previous
Next »
