# Supplementary material for: ADMIRE: analysis and visualization of differential methylation in genomic regions using the Infinium HumanMethylation450 Assay
Source: Epigenetics Chromatin. 2015 Dec 1;8:51. doi: 10.1186/s13072-015-0045-1 (PMC4666223; doi:10.1186/s13072-015-0045-1)
Supplement: Supplementary file 3 — 10.1186/s13072-015-0045-1 ADMIRE documentation. The documentation provides description of all available parameters, input and output files as well as an example analysis of the atrial fibrillation data used in this publication. [file 13072_2015_45_MOESM3_ESM.zip › galaxy-example/index.html]

  


Analysing example datasets - ADMIRE


ADMIRE

- - Home
  - - - Using the web service
      - Analysing example datasets
      - Analysing custom datasets
      - Available parameters- - - Command-line usage
          - Installation
          - HiScan/iScan scanner files
          - Custom input
          - Genomic regions
          - Gene sets
          - Available parameters- - - Output
              - - - MIT License

ADMIRE

- Docs »
- Using the web service »
- Analysing example datasets
- Edit on GitHub

---

We provide two example datasets as shared data that can be used to try ADMIRE online.

1. When navigating the web service of ADMIRE, click on *Shared Data* in the top panel and select *Data Libraries*.
2. Next, choose the *ADMIRE examples* from the available shared data libraries.
3. Check the boxes of one or two sample definition files that you would like to analyse.
4. Now you can perform the preselected *Import to current history* action for selected files by clicking on *Go*. This action will create new datasets in the right panel.
5. Click on *Analyze Data* in the top panel to return to the main site.
6. Choose *admire methylation analysis* from the tools on the left.
7. Select *Operate on a tabular sample definition file* as input mode. The tabular sample definition file should be chosen automatically.
8. As a last step, choose genomic regions of interest and (optional) gene sets.
9. Hit the **Execute** button to see ADMIRE in action.

Next 
 Previous

---

Built with MkDocs using a theme provided by Read the Docs.

GitHub
« Previous
Next »
