# Supplementary material for: ADMIRE: analysis and visualization of differential methylation in genomic regions using the Infinium HumanMethylation450 Assay
Source: Epigenetics Chromatin. 2015 Dec 1;8:51. doi: 10.1186/s13072-015-0045-1 (PMC4666223; doi:10.1186/s13072-015-0045-1)
Supplement: Supplementary file 3 — 10.1186/s13072-015-0045-1 ADMIRE documentation. The documentation provides description of all available parameters, input and output files as well as an example analysis of the atrial fibrillation data used in this publication. [file 13072_2015_45_MOESM3_ESM.zip › galaxy-parameters/index.html]

  


Available parameters - ADMIRE


ADMIRE

- - Home
  - - - Using the web service
      - Analysing example datasets
      - Analysing custom datasets
      - Available parameters- - - Command-line usage
          - Installation
          - HiScan/iScan scanner files
          - Custom input
          - Genomic regions
          - Gene sets
          - Available parameters- - - Output
              - - - MIT License

ADMIRE

- Docs »
- Using the web service »
- Available parameters
- Edit on GitHub

---

**Include quality control report in output**:

If you check this option, a PDF file will be created containing the output of *minfi*'s quality control routines.

**Select a method for input normalization**:

Normalization is used to remove unwanted variation and normalize between arrays. You can choose from five different normalization methods:

1. Functional normalization
2. Noob normalization
3. SWAN normalization
4. Quantile normalization
5. Illumina Genome Studio normalization

Additionaly, you can skip the normalization step by selecting *No normalization - use raw values*.

**Detection p-value threshold for failed probe identification**:

Every probe on the array has a detection p-value assigned, which indicates confidence of the scanner that the detection was correct. A probe will be marked as *failed* in a sample if its detection p-value is higher that the given value.
Together with the

**Failed sample threshold**:

You can subsequently exclude probes that have a certain proportion of failed marks across all samples.

**Q-value cutoff for multiple testing**:

Multiple testing is done automatically by ADMIRE and corrects the test statistic for multiple performed tests. If a certain region remains with a Q-value or FDR below the given value, it will be retained for subsequent analysis, like the gene set enrichment analysis or visualizations.

**Number of additional plots for n best regions**:

The number given here will determine how many visualizations are plotted for significant regions. Importantly, if the number of samples is higher than 100, a heatmap is created. Otherwise, non-proportional bubble plots are plotted.

**Select genomic regions to test**:

Regions selected here will be overlapped with methylation probes and significant different methylated regions will be reported.

By uploading bed files to the work space (right panel), users can give custom regions by adding them to the **custom genomic regions** list.

**Choose gene sets**:

If a gene set is given (either by selecting pre-defined gene sets or uploading a custom gene set), a gene set enrichment analysis is performed by taking all significantly different methylathed regions that are annotated with a gene name and testing them for enrichment in a gene set.
Regions with annotated gene names are *Promoter Regions (2kB)* and *Exons*.

Next 
 Previous

---

Built with MkDocs using a theme provided by Read the Docs.

GitHub
« Previous
Next »
