# Supplementary material for: ADMIRE: analysis and visualization of differential methylation in genomic regions using the Infinium HumanMethylation450 Assay
Source: Epigenetics Chromatin. 2015 Dec 1;8:51. doi: 10.1186/s13072-015-0045-1 (PMC4666223; doi:10.1186/s13072-015-0045-1)
Supplement: Supplementary file 3 — 10.1186/s13072-015-0045-1 ADMIRE documentation. The documentation provides description of all available parameters, input and output files as well as an example analysis of the atrial fibrillation data used in this publication. [file 13072_2015_45_MOESM3_ESM.zip › galaxy-usage/index.html]

  


Analysing custom datasets - ADMIRE


ADMIRE

- - Home
  - - - Using the web service
      - Analysing example datasets
      - Analysing custom datasets
      - Available parameters- - - Command-line usage
          - Installation
          - HiScan/iScan scanner files
          - Custom input
          - Genomic regions
          - Gene sets
          - Available parameters- - - Output
              - - - MIT License

ADMIRE

- Docs »
- Using the web service »
- Analysing custom datasets
- Edit on GitHub

---

Before running your own analysis using the ADMIRE web service, you should have the following files on your local desktop:

- a SampleSheet.csv file and
- file directories named after the assays Sentrix-ID containing two \*.idat files per sample.

To reduce the amount of data to send to the web server, compress the folders containing the \*.idat files. To do this, select all folders and use built-in solutions from Windows or Mac to create a file that ends with

- .tar.gz, .tgz - uses the tar compression together with gzip or
- .tar.bz2, .tbz2, .tar.bzip2

Now register a user by selecting the *User* tab in the top panel of the ADMIRE web page, followed by a click on *Register*. Fill in your email address and choose a password and a public name.
You don't have to wait for a confirmation email and can work right out of the box.

By creating a user account, you'll also be granted access to your personal FTP space on the server. As a next step, upload the compressed idat files into your remote FTP space.
To do so, use any FTP client or Windows Explorers built-in capabilities to connect to the FTP server at ftp://bioinformatics.mpi-bn.mpg.de:

Now drag and drop the SampleSheet.csv and the compressed file you created earlier into the FTP space:

Change back to the web server and use the *Upload Tool* (1) to select the *SampleSheet.csv* file (2) **only** and upload it by clicking on *Execute* (3).

Now choose the *ADMIRE* tool from the toolbar (4) and fill in the file name of the compressed idat files.

As a last step, choose genomic regions of interest and (optional) gene sets (8).
Hit the **Execute** button to see ADMIRE in action (9).

Next 
 Previous

---

Built with MkDocs using a theme provided by Read the Docs.

GitHub
« Previous
Next »
