# Supplementary material for: ADMIRE: analysis and visualization of differential methylation in genomic regions using the Infinium HumanMethylation450 Assay
Source: Epigenetics Chromatin. 2015 Dec 1;8:51. doi: 10.1186/s13072-015-0045-1 (PMC4666223; doi:10.1186/s13072-015-0045-1)
Supplement: Supplementary file 3 — 10.1186/s13072-015-0045-1 ADMIRE documentation. The documentation provides description of all available parameters, input and output files as well as an example analysis of the atrial fibrillation data used in this publication. [file 13072_2015_45_MOESM3_ESM.zip › hiscan/index.html]

  


HiScan/iScan scanner files - ADMIRE


ADMIRE

- - Home
  - - - Using the web service
      - Analysing example datasets
      - Analysing custom datasets
      - Available parameters- - - Command-line usage
          - Installation
          - HiScan/iScan scanner files
          - Custom input
          - Genomic regions
          - Gene sets
          - Available parameters- - - Output
              - - - MIT License

ADMIRE

- Docs »
- Command-line usage »
- HiScan/iScan scanner files
- Edit on GitHub

---

The default output of HumanMethylation450 BeadChip compatible scanner systems consists of

- a SampleSheet.csv file and
- file directories named after the Chips Sentrix-ID containing two \*.idat files per sample.

To use the files generated by the scanner system with ADMIRE, all file directories have to be compressed (e.g. by running `tar -zcvf compressFileName.tar.gz folderToCompress`).

ADMIRE can then be called with `admire -c SampleSheet.csv -z compressFileName.tar.gz`

*Hint*: ADMIRE can also read files ending on \*.tar.gz, \*.tgz or \*.tbz2.

Next 
 Previous

---

Built with MkDocs using a theme provided by Read the Docs.

GitHub
« Previous
Next »
