# Supplementary material for: ADMIRE: analysis and visualization of differential methylation in genomic regions using the Infinium HumanMethylation450 Assay
Source: Epigenetics Chromatin. 2015 Dec 1;8:51. doi: 10.1186/s13072-015-0045-1 (PMC4666223; doi:10.1186/s13072-015-0045-1)
Supplement: Supplementary file 3 — 10.1186/s13072-015-0045-1 ADMIRE documentation. The documentation provides description of all available parameters, input and output files as well as an example analysis of the atrial fibrillation data used in this publication. [file 13072_2015_45_MOESM3_ESM.zip › install/index.html]

  


Installation - ADMIRE


ADMIRE

- - Home
  - - - Using the web service
      - Analysing example datasets
      - Analysing custom datasets
      - Available parameters- - - Command-line usage
          - Installation
          - HiScan/iScan scanner files
          - Custom input
          - Genomic regions
          - Gene sets
          - Available parameters- - - Output
              - - - MIT License

ADMIRE

- Docs »
- Command-line usage »
- Installation
- Edit on GitHub

---

Make sure all prerequisites are met:

- comb-p
- bedtools
- R/Rscript
- python - numpy
- python - matplotlib

Install admire locally by running:

```
git clone https://github.molgen.mpg.de/loosolab/admire.git
export PATH=$PATH:dir/to/admire/src
```

Next 
 Previous

---

Built with MkDocs using a theme provided by Read the Docs.

GitHub
« Previous
Next »
