# Supplementary material for: ADMIRE: analysis and visualization of differential methylation in genomic regions using the Infinium HumanMethylation450 Assay
Source: Epigenetics Chromatin. 2015 Dec 1;8:51. doi: 10.1186/s13072-015-0045-1 (PMC4666223; doi:10.1186/s13072-015-0045-1)
Supplement: Supplementary file 3 — 10.1186/s13072-015-0045-1 ADMIRE documentation. The documentation provides description of all available parameters, input and output files as well as an example analysis of the atrial fibrillation data used in this publication. [file 13072_2015_45_MOESM3_ESM.zip › license/index.html]

  


MIT License - ADMIRE


ADMIRE

- - Home
  - - - Using the web service
      - Analysing example datasets
      - Analysing custom datasets
      - Available parameters- - - Command-line usage
          - Installation
          - HiScan/iScan scanner files
          - Custom input
          - Genomic regions
          - Gene sets
          - Available parameters- - - Output
              - - - MIT License

ADMIRE

- Docs »
- MIT License
- Edit on GitHub

---

Copyright (c) 2015 Jens Preussner and Mario Looso

Permission is hereby granted, free of charge, to any person obtaining a copy of this software and associated documentation files (the "Software"), to deal in the Software without restriction, including without limitation the rights to use, copy, modify, merge, publish, distribute, sublicense, and/or sell copies of the Software, and to permit persons to whom the Software is furnished to do so, subject to the following conditions:

The above copyright notice and this permission notice shall be included in all copies or substantial portions of the Software.

THE SOFTWARE IS PROVIDED "AS IS", WITHOUT WARRANTY OF ANY KIND, EXPRESS OR IMPLIED, INCLUDING BUT NOT LIMITED TO THE WARRANTIES OF MERCHANTABILITY, FITNESS FOR A PARTICULAR PURPOSE AND NONINFRINGEMENT. IN NO EVENT SHALL THE AUTHORS OR COPYRIGHT HOLDERS BE LIABLE FOR ANY CLAIM, DAMAGES OR OTHER LIABILITY, WHETHER IN AN ACTION OF CONTRACT, TORT OR OTHERWISE, ARISING FROM, OUT OF OR IN CONNECTION WITH THE SOFTWARE OR THE USE OR OTHER DEALINGS IN THE SOFTWARE.

Previous

---

Built with MkDocs using a theme provided by Read the Docs.

GitHub
« Previous
