# Supplementary material for: ADMIRE: analysis and visualization of differential methylation in genomic regions using the Infinium HumanMethylation450 Assay
Source: Epigenetics Chromatin. 2015 Dec 1;8:51. doi: 10.1186/s13072-015-0045-1 (PMC4666223; doi:10.1186/s13072-015-0045-1)
Supplement: Supplementary file 3 — 10.1186/s13072-015-0045-1 ADMIRE documentation. The documentation provides description of all available parameters, input and output files as well as an example analysis of the atrial fibrillation data used in this publication. [file 13072_2015_45_MOESM3_ESM.zip › output/index.html]

  


Output - ADMIRE


ADMIRE

- - Home
  - - - Using the web service
      - Analysing example datasets
      - Analysing custom datasets
      - Available parameters- - - Command-line usage
          - Installation
          - HiScan/iScan scanner files
          - Custom input
          - Genomic regions
          - Gene sets
          - Available parameters- - - Output
                - Files in the excel subdirectory
                - Files in the visualization subdirectory
                - Files in the normalized subdirectory
                - Files in the results subdirectory
                - Files in the geneset-enrichment subdirectory
              - - - MIT License

ADMIRE

- Docs »
- Output
- Edit on GitHub

---

ADMIRE creates a number of output directories and files that are described below:

#### Files in the excel subdirectory

This subdirectory contains csv files for each combination of sample group comparison (e.g. case-vs-control) and genomic region (e.g. promoters), with information about the genomic feature, its genomic location as well as p- and q-values of the sample groups. In addition to a file with all regions passing the q-value threshold, a file containing all regions is also present.

#### Files in the visualization subdirectory

This subdirectory contains files for visualization with IGV. General files, like the genomic location of all Illumina probes, as well as the genomics regions used during analysis, are located in the `annotation-tracks` subfolder. Data specific files are located in the `data-tracks` folder. Here, you can find information per sample-group comparison (e.g. case-vs-control), information on significantly altered probe methylation (`control-case.igv`), as well as significantly altered genomic regions in BED format.
Additionally, publication-ready images are stored in region-specific subdirectories.

#### Files in the normalized subdirectory

When using on the command line, the `normalized` subdirectory contains two matrices per normalization method, one with (normalized) beta values, the other with (normalized) m values. Each row corresponds to a single Illumina probe, each column represents a sample.

#### Files in the results subdirectory

When using on the command line, the `results` subdirectory contains intermediate result files, like the output from statistical testing (`control-case.pvals.bed`) or results from combining p-values (`comb-p` subdirectory).

#### Files in the geneset-enrichment subdirectory

The `geneset_enrichment` subdirectory contains two tables for each sample group vs. genomic region combination. The first table (`control-case-region-genesets_ranked.txt`) gives per gene set information on the size, ES, NES and Q-value. The second table (`control-case-region-geneset_genes.txt`) contains information like the position in the ranked list and the running ES at that position for each gene vs. gene set combination. Additionally, a PDF file is created for each gene set, showing its enrichment plot.

Next 
 Previous

---

Built with MkDocs using a theme provided by Read the Docs.

GitHub
« Previous
Next »
