# Supplementary material for: ADMIRE: analysis and visualization of differential methylation in genomic regions using the Infinium HumanMethylation450 Assay
Source: Epigenetics Chromatin. 2015 Dec 1;8:51. doi: 10.1186/s13072-015-0045-1 (PMC4666223; doi:10.1186/s13072-015-0045-1)
Supplement: Supplementary file 3 — 10.1186/s13072-015-0045-1 ADMIRE documentation. The documentation provides description of all available parameters, input and output files as well as an example analysis of the atrial fibrillation data used in this publication. [file 13072_2015_45_MOESM3_ESM.zip › parameters/index.html]

  


Available parameters - ADMIRE


ADMIRE

- - Home
  - - - Using the web service
      - Analysing example datasets
      - Analysing custom datasets
      - Available parameters- - - Command-line usage
          - Installation
          - HiScan/iScan scanner files
          - Custom input
          - Genomic regions
          - Gene sets
          - Available parameters- - - Output
              - - - MIT License

ADMIRE

- Docs »
- Command-line usage »
- Available parameters
- Edit on GitHub

---

Here we list all current parameters for command-line usage and their explanation:

```
Usage: admire [options]

Available options:
-c | Comma separated sample definition file (SampleSheet.csv)
-s | Tab separated sample definition file (design.txt)
-z | Compressed input of idat files (requires -c).
-e | Create quality control report in PDF
-r | Region file in bed format (regions.bed), use multiple -r parameters to calculate for multiple region files
-p | Detection p-value to exclude probes prior to analysis (0.01)
-t | Exclude probes where more than t% samples failed according to the detection p-value. (0.4)
-n | Normalization method (fn,swan,noob,illumina,raw,quantile)
-b | In case of functional normalization, skip noob background correction step
-d | In case of noob or functional normalization, skip dye correction step
-f | In case of quantile normalization, skip fixing outliers prior to analysis
-l | In case of quantile normalization, label samples as bad if their median signals are below a given value (10.5)
-m | In case of quantile normalization, remove bad samples
-q | Q-value cutoff for multiple testing correction (0.05)
-i | Render advanced plots for the best i regions (20)
-g | Gene set file for enrichment analysis, use multiple -g parameters to calculate enrichment over many gene sets
-o | tar-gz compress output into file given
-h | shows this help message
-v | shows version information

Options -c and -s are mutually exclusive.
```

Next 
 Previous

---

Built with MkDocs using a theme provided by Read the Docs.

GitHub
« Previous
Next »
