# Supplementary material for: ADMIRE: analysis and visualization of differential methylation in genomic regions using the Infinium HumanMethylation450 Assay
Source: Epigenetics Chromatin. 2015 Dec 1;8:51. doi: 10.1186/s13072-015-0045-1 (PMC4666223; doi:10.1186/s13072-015-0045-1)
Supplement: Supplementary file 3 — 10.1186/s13072-015-0045-1 ADMIRE documentation. The documentation provides description of all available parameters, input and output files as well as an example analysis of the atrial fibrillation data used in this publication. [file 13072_2015_45_MOESM3_ESM.zip › regions/index.html]

  


Genomic regions - ADMIRE


ADMIRE

- - Home
  - - - Using the web service
      - Analysing example datasets
      - Analysing custom datasets
      - Available parameters- - - Command-line usage
          - Installation
          - HiScan/iScan scanner files
          - Custom input
          - Genomic regions
          - Gene sets
          - Available parameters- - - Output
              - - - MIT License

ADMIRE

- Docs »
- Command-line usage »
- Genomic regions
- Edit on GitHub

---

Custom genomic regions should be provided in BED format and can be given by `admire -r regions1.bed -r regions2.bed ...`.

*Hint*: Use multiple `-r` parameters to analyse more than one region at a time.

*Hint*: The BED format is described here.

To enable the Gene Set Enrichment Analysis for a certain bed file, include a *gene\_name* property in column 4 of the bed file:

```
chr1    213941196   213942363   gene_name=gene1
chr1    213942363   213943530   gene_name=gene2
chr1    213943530   213944697   gene_name=gene3
```

Next 
 Previous

---

Built with MkDocs using a theme provided by Read the Docs.

GitHub
« Previous
Next »
