# Supplementary material for: ADMIRE: analysis and visualization of differential methylation in genomic regions using the Infinium HumanMethylation450 Assay
Source: Epigenetics Chromatin. 2015 Dec 1;8:51. doi: 10.1186/s13072-015-0045-1 (PMC4666223; doi:10.1186/s13072-015-0045-1)
Supplement: Supplementary file 3 — 10.1186/s13072-015-0045-1 ADMIRE documentation. The documentation provides description of all available parameters, input and output files as well as an example analysis of the atrial fibrillation data used in this publication. [file 13072_2015_45_MOESM3_ESM.zip › index.html]

  


ADMIRE


ADMIRE

- - Home
    - Welcome to the ADMIRE documentation
    - Overview and Objective
    - Features
    - How to cite?
    - Contribute
    - Support
    - License
  - - - Using the web service
      - Analysing example datasets
      - Analysing custom datasets
      - Available parameters- - - Command-line usage
          - Installation
          - HiScan/iScan scanner files
          - Custom input
          - Genomic regions
          - Gene sets
          - Available parameters- - - Output
              - - - MIT License

ADMIRE

- Docs »
- Home
- Edit on GitHub

---

# Welcome to the ADMIRE documentation

ADMIRE is a semi-automatic analysis pipeline and visualization tool for Infinium HumanMethylation450K Chips.

Use ADMIRE online: bioinformatics.mpi-bn.mpg.de

## Overview and Objective

DNA methylation at cytosine nucleotides constitutes epigenetic gene regulation impacting cellular development and the stage of a disease. Besides whole genome bisulfit sequencing, Illumina HumanMethylation450K Assays represent a versatile and cost-effective tool to investigate changes of methylation patterns at CpG sites.
ADMIRE was developed as an open source, semi-automatic analysis pipeline and visualization tool for Illumina HumanMethylation450K Assays.

## Features

- Automatic filtering and normalization
- Statistical testing and multiple testing correction
- Supports arbitrary number of samples and sample groups
- Differential methylation analysis on pre-calculated and individual genomic regions
- Provides ready-to-plug-in files for genome browsers (like IGV)
- Provides publication-ready figures for the most differentially methylated regions
- Performs gene set enrichment analysis on predefined and individual gene sets

## How to cite?

Please cite the paper describing ADMIRE when using the web service or command line version in your research:

Preussner J, Bayer J, Kuenne C and Looso M. ADMIRE: Analysis and visualization of differential methylation in genomic regions using the Infinium HumanMethylation450 Assay. *Epigenetics & Chromatin* (**2015**).

## Contribute

- Issue Tracker: https://github.molgen.mpg.de/loosolab/admire/issues
- Source Code: https://github.molgen.mpg.de/loosolab/admire

## Support

If you are having issues, please feel free to send an e-mail to Jens Preußner (jens.preussner@mpi-bn.mpg.de).

## License

The project is licensed under the MIT license.

Next

---

Built with MkDocs using a theme provided by Read the Docs.

GitHub
Next »
