# Supplementary figures and images for: ADMIRE: analysis and visualization of differential methylation in genomic regions using the Infinium HumanMethylation450 Assay
Source: Epigenetics Chromatin. 2015 Dec 1;8:51. doi: 10.1186/s13072-015-0045-1 (PMC4666223; doi:10.1186/s13072-015-0045-1)

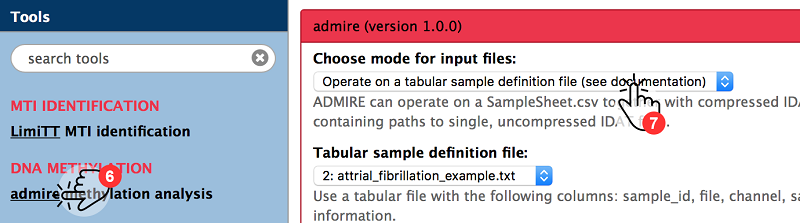

Supplement: Supplementary file 3 — 10.1186/s13072-015-0045-1 ADMIRE documentation. The documentation provides description of all available parameters, input and output files as well as an example analysis of the atrial fibrillation data used in this publication. [file 13072_2015_45_MOESM3_ESM.zip › img/admire.png]

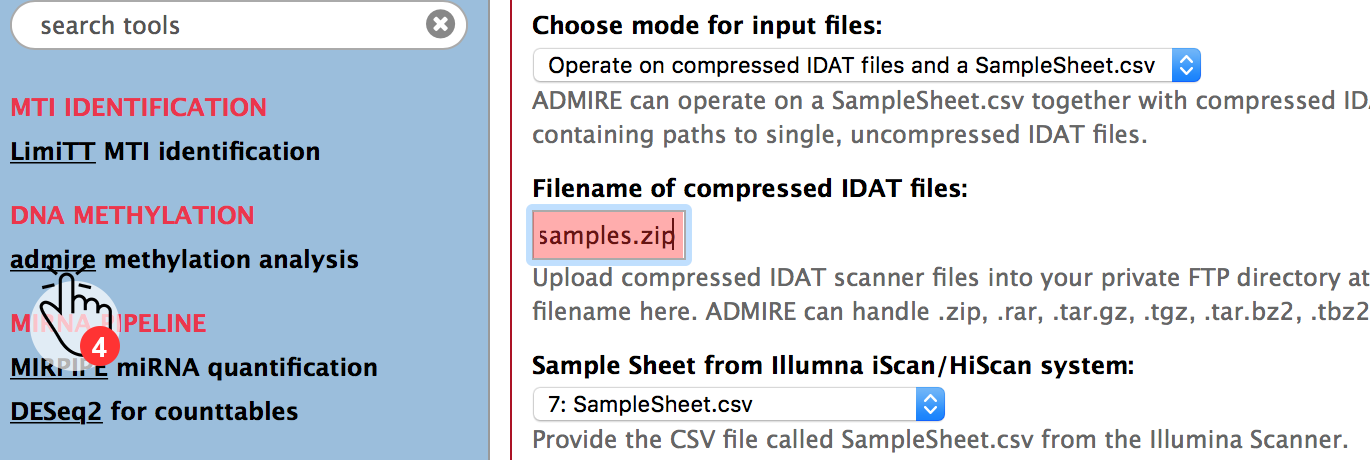

Supplement: Supplementary file 3 — 10.1186/s13072-015-0045-1 ADMIRE documentation. The documentation provides description of all available parameters, input and output files as well as an example analysis of the atrial fibrillation data used in this publication. [file 13072_2015_45_MOESM3_ESM.zip › img/admire2.png]

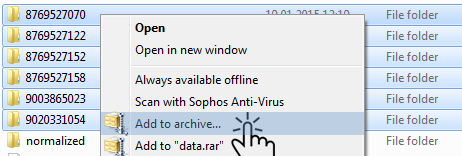

Supplement: Supplementary file 3 — 10.1186/s13072-015-0045-1 ADMIRE documentation. The documentation provides description of all available parameters, input and output files as well as an example analysis of the atrial fibrillation data used in this publication. [file 13072_2015_45_MOESM3_ESM.zip › img/compress.png]

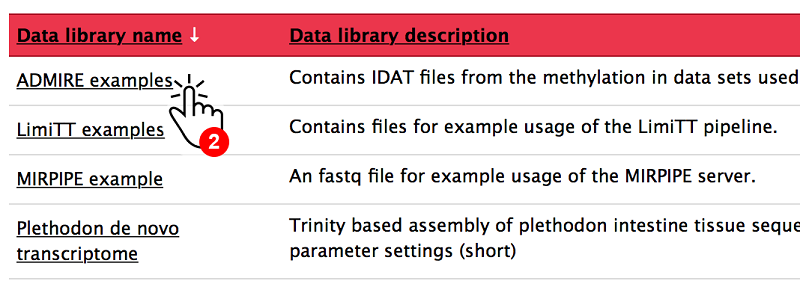

Supplement: Supplementary file 3 — 10.1186/s13072-015-0045-1 ADMIRE documentation. The documentation provides description of all available parameters, input and output files as well as an example analysis of the atrial fibrillation data used in this publication. [file 13072_2015_45_MOESM3_ESM.zip › img/data-libraries.png]

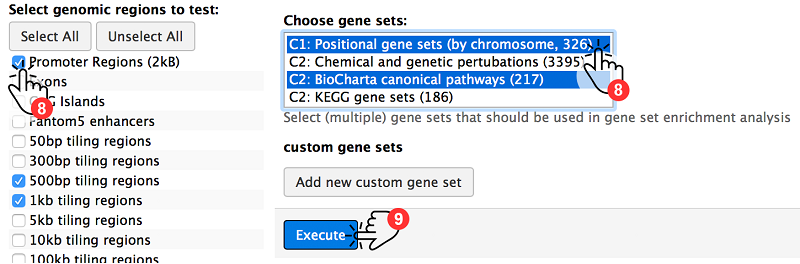

Supplement: Supplementary file 3 — 10.1186/s13072-015-0045-1 ADMIRE documentation. The documentation provides description of all available parameters, input and output files as well as an example analysis of the atrial fibrillation data used in this publication. [file 13072_2015_45_MOESM3_ESM.zip › img/execute.png]

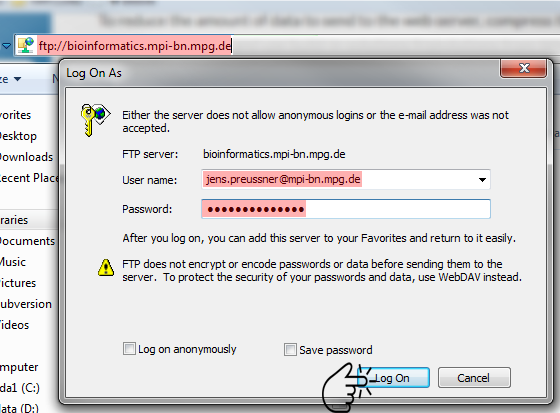

Supplement: Supplementary file 3 — 10.1186/s13072-015-0045-1 ADMIRE documentation. The documentation provides description of all available parameters, input and output files as well as an example analysis of the atrial fibrillation data used in this publication. [file 13072_2015_45_MOESM3_ESM.zip › img/ftp.png]

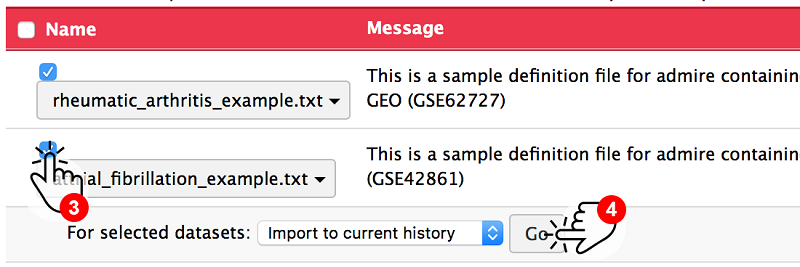

Supplement: Supplementary file 3 — 10.1186/s13072-015-0045-1 ADMIRE documentation. The documentation provides description of all available parameters, input and output files as well as an example analysis of the atrial fibrillation data used in this publication. [file 13072_2015_45_MOESM3_ESM.zip › img/import.png]

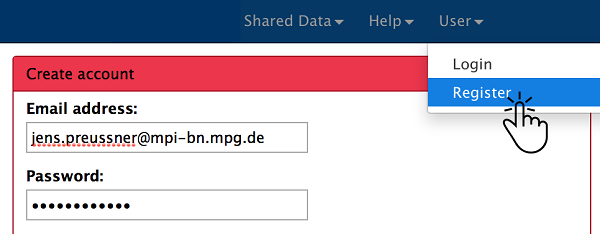

Supplement: Supplementary file 3 — 10.1186/s13072-015-0045-1 ADMIRE documentation. The documentation provides description of all available parameters, input and output files as well as an example analysis of the atrial fibrillation data used in this publication. [file 13072_2015_45_MOESM3_ESM.zip › img/register.png]

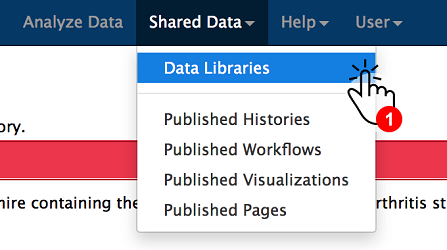

Supplement: Supplementary file 3 — 10.1186/s13072-015-0045-1 ADMIRE documentation. The documentation provides description of all available parameters, input and output files as well as an example analysis of the atrial fibrillation data used in this publication. [file 13072_2015_45_MOESM3_ESM.zip › img/shared-data.png]

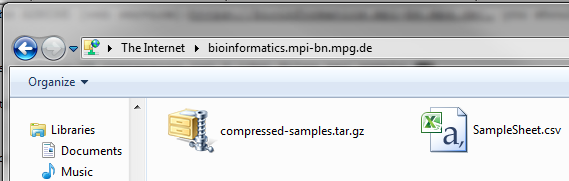

Supplement: Supplementary file 3 — 10.1186/s13072-015-0045-1 ADMIRE documentation. The documentation provides description of all available parameters, input and output files as well as an example analysis of the atrial fibrillation data used in this publication. [file 13072_2015_45_MOESM3_ESM.zip › img/upload.png]

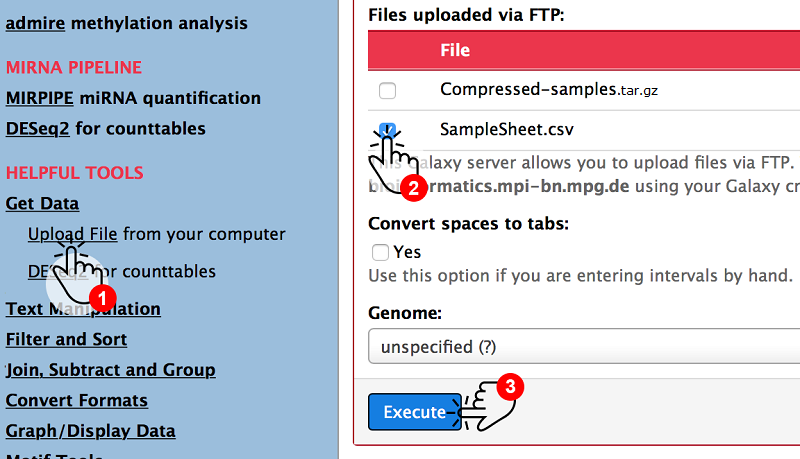

Supplement: Supplementary file 3 — 10.1186/s13072-015-0045-1 ADMIRE documentation. The documentation provides description of all available parameters, input and output files as well as an example analysis of the atrial fibrillation data used in this publication. [file 13072_2015_45_MOESM3_ESM.zip › img/upload2.png]

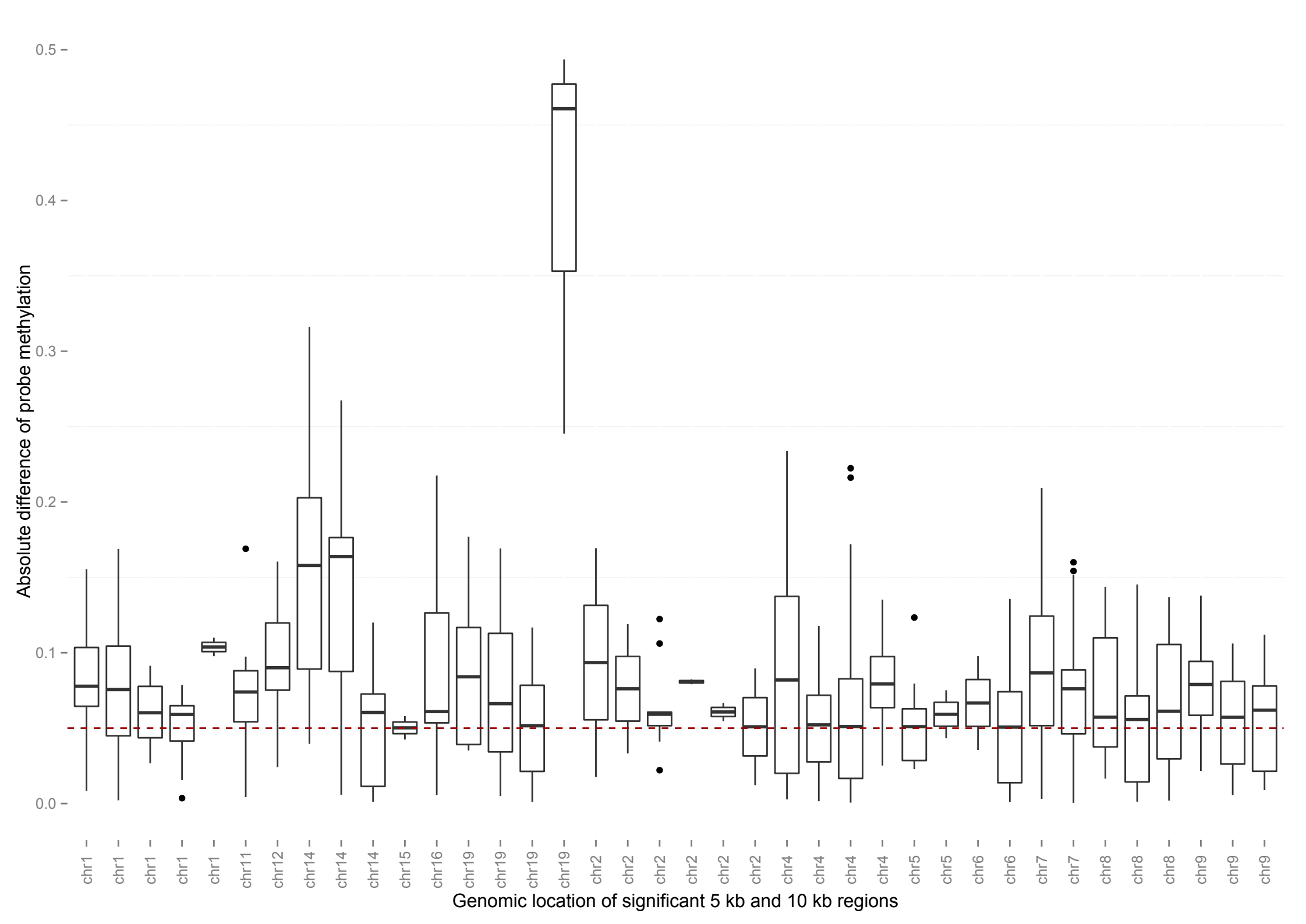

Supplement: Supplementary file 5 — 10.1186/s13072-015-0045-1 Absolute difference of methylation in 5 and 10 kB tiling regions in atrial fibrillation reported by ADMIRE. Boxplots give information about the magnitude of methylation change. [file 13072_2015_45_MOESM5_ESM.pdf]

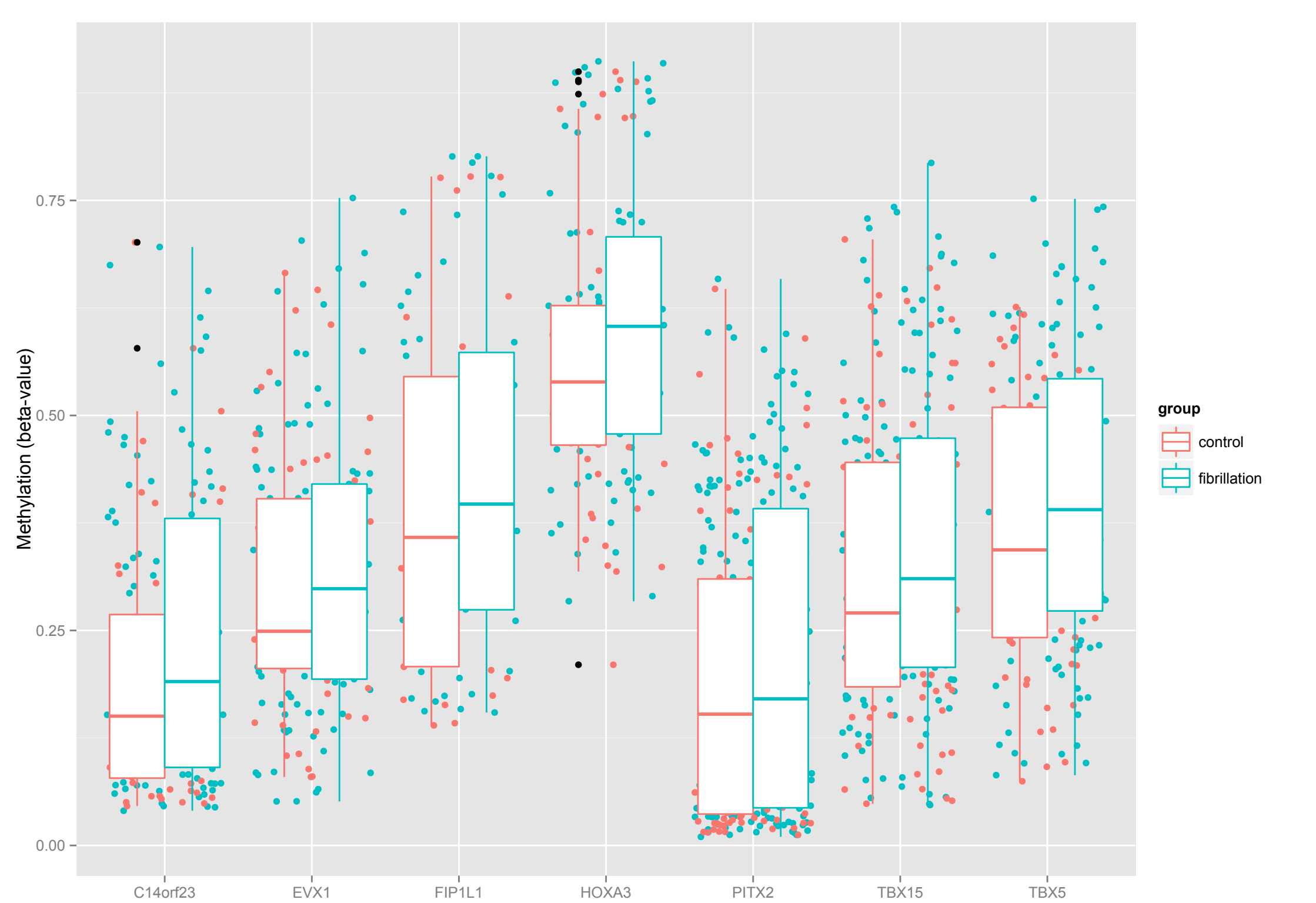

Supplement: Supplementary file 6 — 10.1186/s13072-015-0045-1 Beta values of protein coding genes with significantly differential methylation between patients with atrial fibrillation and healthy individuals. [file 13072_2015_45_MOESM6_ESM.pdf]

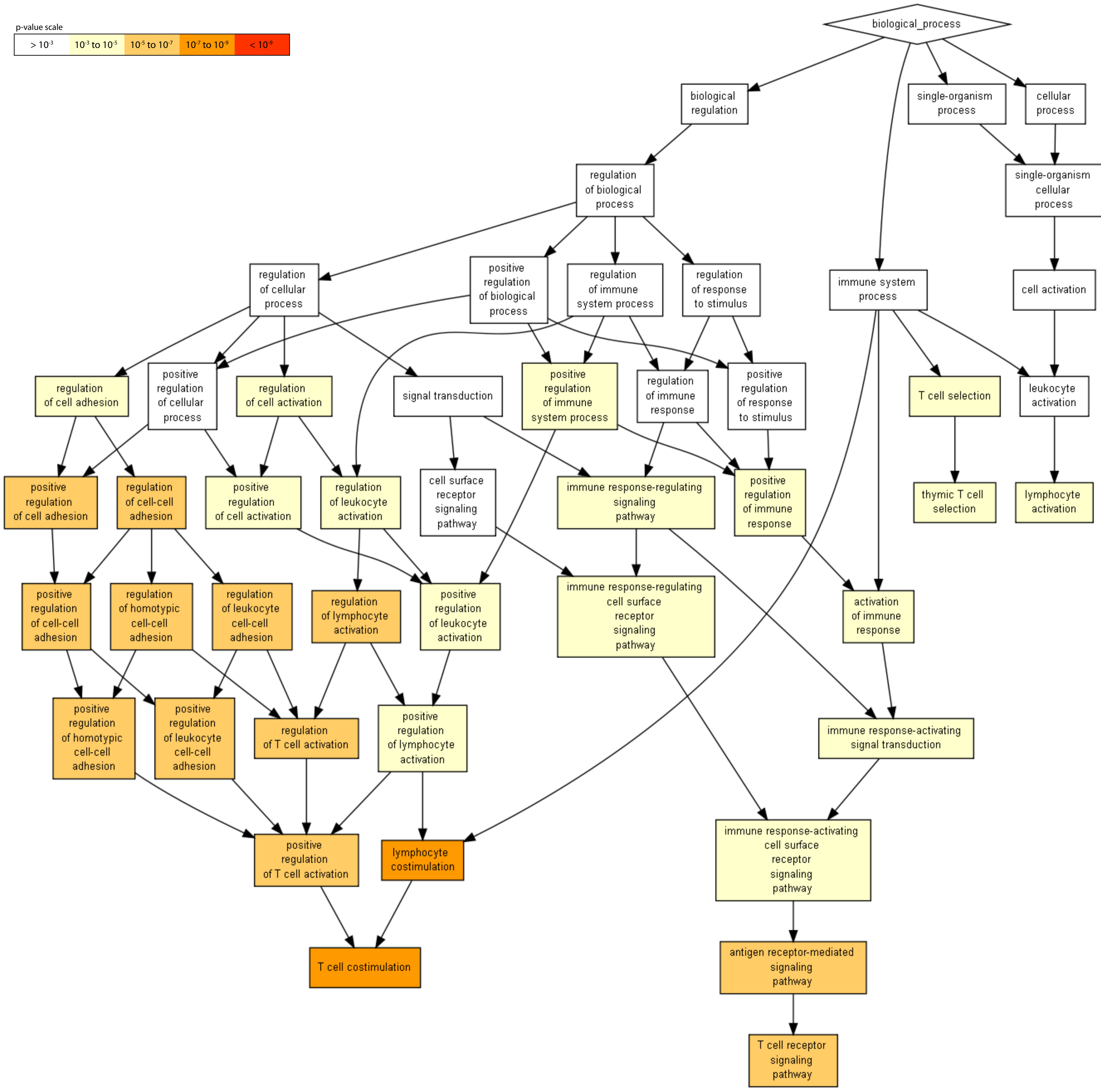

Supplement: Supplementary file 8 — 10.1186/s13072-015-0045-1 Enriched functional GO-Terms of genes with higher methylation values in RA. Background color codes for p values in the following way: >10–3 (white), 10–3 to 10–5 (light yellow) and 10–7 to 10–9 (orange). [file 13072_2015_45_MOESM8_ESM.pdf]
